# Transcriptome Analysis Deciphers the Underlying Molecular Mechanism of Peanut Lateral Branch Angle Formation Using Erect Branching Mutant

**DOI:** 10.3390/genes15101348

**Published:** 2024-10-21

**Authors:** Liangqiong He, Conghui Yu, Guanghao Wang, Lei Su, Xin Xing, Tiantian Liu, Zhipeng Huang, Han Xia, Shuzhen Zhao, Zhongkui Gao, Xingjun Wang, Chuanzhi Zhao, Zhuqiang Han, Jiaowen Pan

**Affiliations:** 1 Guangxi Academy of Agricultural Sciences, Nanning 530007, China; heliangqiong@163.com (L.H.); zhjxc15@163.com (Z.H.); gaozhongkui21@163.com (Z.G.); 2 Shandong International Joint Laboratory of Agricultural Germplasm Resources Innovation, Institute of Crop Germplasm Resources (Institute of Biotechnology), Shandong Academy of Agricultural Sciences, Jinan 250100, China; 17866713650@163.com (C.Y.); wgh90325@126.com (G.W.); coldxia@126.com (H.X.); zhaoshuzhen51@126.com (S.Z.); xingjunw@hotmail.com (X.W.); chuanzhiz@126.com (C.Z.); 3 College of Life Sciences, Shandong Normal University, Jinan 250014, China; 4 Kenli District Agricultural Development Service Center, Dongying 257500, China; 18561208026@163.com; 5 Weihai City Agricultural and Rural Affairs Service Center, Weihai 264200, China; xingxinsdau@163.com (X.X.); Liutiantian356@126.com (T.L.)

**Keywords:** peanut, lateral branch angle (LBA), *eg06g*, auxin, RNA-seq

## Abstract

**Background** The growth habit (GH), also named the branching habit, is an important agronomic trait of peanut and mainly determined by the lateral branch angle (LBA). The branching habit is closely related to peanut mechanized farming, pegging, yield, and disease management. **Objectives** However, the molecular basis underlying peanut LBA needs to be uncovered. **Methods** In the present study, an erect branching peanut mutant, *eg06g*, was obtained via ^60^Co γ-ray-radiating mutagenesis of a spreading-type peanut cultivar, Georgia-06G (G06G). RNA-seq was performed to compare the transcriptome variation of the upper sides and lower sides of the lateral branch of *eg06g* and G06G. **Results** In total, 4908 differentially expressed genes (DEGs) and 5833 DEGs were identified between *eg06g* and G06G from the lower sides and upper sides of the lateral branch, respectively. GO, KEGG, and clustering enrichment analysis indicated that the carbohydrate metabolic process, cell wall organization or biogenesis, and plant hormone signal transduction were mainly enriched in *eg06g*. **Conclusions** Further analysis showed that the genes involved in starch biosynthesis were upregulated in *eg06g*, which contributed to amyloplast sedimentation and gravity perception. Auxin homeostasis and transport-related genes were found to be upregulated in *eg06g*, which altered the redistribution of auxin in *eg06g* and in turn triggered apoplastic acidification and activated cell wall modification-related enzymes, leading to tiller angle establishment through the promotion of cell elongation at the lower side of the lateral branch. In addition, cytokinin and GA also demonstrated synergistic action to finely regulate the formation of peanut lateral branch angles. Collectively, our findings provide new insights into the molecular regulation of peanut LBA and present genetic materials for breeding peanut cultivars with ideotypes.

## 1. Introduction

Peanut is an importantly economical oilseed crop and cultivated in more than 100 countries, supplying 20% of global cooking oil and 11% of protein production [1]. Cultivated peanut (*Arachis hypogaea* L.) is an allotetraploid (AABB genome, 2n = 4x = 40) and putatively originates from the natural hybridization between two wild diploid progenitors, *Arachis duranensis* (AA genome, 2n = 2x = 20) and *A. ipaënsis* (BB genome, 2n = 2x = 20) [2]. Cultivated peanut has been domesticated for more than 3500 years, and the growth habit was a visibly domestication-related morphological modification, which converted from the prostrate growth habit of the wild diploid species to the erect or bunch habit of the cultivated varieties [3]. 

The growth habit, also named the branching habit, is a critical agronomic trait of peanut and mainly determined by the lateral branch angle (LBA). More and more studies show that the branching habit is closely associated with peanut mechanical cultivation, flowering, pegging and pod formation, pod maturation, pod yield, and disease management [4]. For domesticated peanut, the branching habit can be divided into four categories: prostrate, spreading, bunch, and erect [5]. According to the index of plant type (IOPT), the peanut branching habit is also classified into three types. The IOPT of spreading type is around 2.0, the semi-spreading type is around 1.5, and the erect type is about 1.1–1.2 [6]. Erect branching peanuts, with compact plant types, are usually suitable for high-density planting, and the pod distribution is mainly concentrated at the base of the main stem. In contrast, the spreading and bunch cultivars occupy larger spreading areas and have a scattered pod distribution [6,7].

Despite the agronomic importance of the branching habit of peanut, both the inheritance and molecular mechanism that controls this trait has not been evidently illuminated. A single gene, two genes, multiple genes, and genic–cytoplasmic interaction have been proposed to control the branching habit in peanut by different research groups [8]. Recent research showed that the bunching trait was controlled by a single gene without a cytoplasmic effect [5]. Several QTLs have been mapped for controlling LBA in peanuts. Shirasawa et al. detected two QTLs, *qAB05.1* and *qAB07.2*, for LBA on LG05.1 and LG07.2, with 11.9% and 23.2% PVE (percentage of variance explained), respectively [9]. Using advanced backcross population, six QTLs for LBA were detected and together explained 46.1% of PVE [10]. Li et al. detected six QTLs associated with LBA and explained 5.02–21.87% of PVE using a recombinant inbred (RIL) population [6]. Using an F_2:3_ population, Kayam et al. (2017) mapped the bunching habit-controlling gene at a 1.1 Mb region on linkage group B5 [5]. Through the BSA-seq and traditional QTL approach, a major gene-controlling LBA was mapped at the region of Chr15: 157.42–157.56 Mb, and nine genes were housed in this region [8]. Using phenotypic recombination BSA/BSR (PR-BSA/BSR), the LBA-related locus was mapped to a 6.82 Mb region of Chr15: 101.74–108.56 Mb [11]. Using RAD-seq, two major QTLs for growth habit were identified in the region of Chr15: 156.78–157.01 Mb and Chr06: 111.94–111.98 Mb [12].

Shoot gravitropism is a crucial determinant of branch angles, which includes gravity perception, signal transduction, auxin asymmetric distribution, and differential growth of abaxial and adaxial sides [13]. The auxin and auxin transport play a central role in this sophisticated process [14]. Multiple genes involved in regulating branch angle have been identified in many plants over the past decade [14]. The rice *lazy1* mutant presents prostrate growth habit, and it regulates rice shoot gravitropism and tiller angle via regulating the asymmetric auxin distribution [14]. AtLAZY, ZmLA1, and CsLAZY1 are the functional ortholog of LAZY1 in *Arabidopsis*, maize, and tea plants, respectively, and mediate shoot gravitropism through regulating auxin transport and signaling [15,16,17]. In response to gravistimulation, the HSFA2D is responsible for inducing asymmetric auxin distribution via activating the expression of *LAZY1*, which in turn induces the asymmetric expression of *WOX6* and *WOX11* and subsequently to regulate the rice tiller angle [13]. OsHOX1 and OsHOX28 acts upstream of HSFA2D to regulate tiller angle establishment through the modulation of the HSFA2D-LA1 pathway and auxin content [18]. TAC1 (tiller angle control 1) is known to promote the horizontal growth of branches, and the mutant of *AtTAC1*, *OsTAC1*, and *PpeTAC1* showed more vertical branch growth angles in *Arabidopsis*, rice, and peach, respectively [19,20]. During gravitropic responses, GA and GA signaling showed asymmetric distribution. At the lower side, high GA levels benefit the stabilization of PIN2 at the lower side of the root and thus contribute to asymmetric auxin flow and distribution for gravitropic bending. During this process, GA acts as a vital part of the complex network, consolidating asymmetric auxin action [21]. Cytokinin promotes shoot branching via mediating accumulation of the PIN3, PIN4, and PIN7 auxin transporters [22]. Strigolactones (SLs) regulate the rice tiller angle through attenuating shoot gravitropism by inhibiting auxin biosynthesis [23]. These phytohormones are involved in regulation shoot branching via directly or indirectly controlling auxin transport and biosynthesis [21,22,23]. 

In this study, an erect branching peanut mutant *eg06g* was obtained by irradiating a spreading-type peanut cultivar, G06G. To verify whether the phytohormones, such as auxin and cytokinin, are also involved in regulating the LBA of peanuts, the upper sides and lower sides of the lateral branch of *eg06g* and G06G were selected for comparative transcriptome analysis. Our objectives were to elucidate the molecular mechanism of branching habit in *eg06g* and provide genetic materials for breeding peanut cultivars with ideotypes.

## 2. Materials and Methods

### 2.1. Acquisition of the eg06g Mutant

A mutant library was constructed via ^60^Co γ-ray radiating G06G, which is a spreading-type peanut cultivar and developed at the University of Georgia, Coastal Plain Experiment Station, Tifton, GA, USA. In M2 generation, a line with an erect branching habit was identified, whose mutant phenotype could be stably inherited in the next generation. After two generations of self-crossing, an erect branching peanut mutant was obtained and defined as erect branching G06G (*eg06g*). 

### 2.2. Planting Conditions and Sampling Methods 

**Figure 1:**
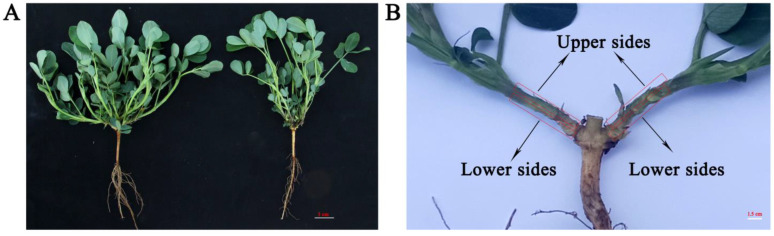
The phenotypes of *eg06g* and G06G. (**A**) The phenotypes of *eg06g* and G06G at 25 days after planting (DAP). (**B**) Schematic illustration of the upper sides and the lower sides of the lateral branch base; the red box highlights the collection location for RNA-seq.

The M6 seeds of *eg06g* and its wild cultivar G06G were planted on the experimental farm of the Shandong Academy of Agricultural Sciences in Jinan. The planting pattern was based on previous studies [24]. About 100 individual plants of G06G and *eg06g* were planted. The morphology and architecture of LBA formed about twenty-five days after planting (DAP). The average LBA of G06G was 83.7° and was significantly larger than that of *eg06g* (LBA 36.4°). As shown in Figure 1A, the upper sides and the lower sides of the lateral branch base from *eg06g* and G06G were sampled and were named EG06Gup, EG06GDown, G06Gup, and G06GDown. Then, the samples were frozen in liquid nitrogen immediately and stored at −80 °C for RNA extraction. A sample was collected from 10 individual plants and three independent biological replicates were performed. 

### 2.3. Library Construction and RNA Sequencing

The total RNA of each sample was extracted and quality tested as described previously [25], and twelve libraries were constructed and sequenced. The RNA of each sample was denatured and enriched by oligo (dT)-attached magnetic beads, and then fragmented in a cleaved reaction system. Using a random hexamer primer, the first-strand cDNA and second-strand cDNA were synthesized sequentially via reverse transcription. The double-stranded cDNA fragments were subjected to end-repair, and sequencing adaptors were connected to the 3′ end. Using specific primers, the repaired cDNA fragments were amplified by PCR, and the products were denatured to single-stranded PCR products. Using a bridge primer, single-stranded PCR products were cyclized to produce a single-stranded cyclic DNA library. Then, sequencing was performed using the BGISEQ-500 platform (BGISEQ-500) (MGI Tech Co., Ltd., Shenzhen, China) at the Beijing Genomics Institute (BGI). 

### 2.4. Data Analysis

Using SOAPnuke, the sequencing data were filtered, and the sequencing adapter and low-quality base were removed. Clean reads were obtained and aligned with the peanut reference genome sequence of *Arachis hypogaea* cv. Tifrunner (Tifrunner.gnm2.J5K5-data.legumeinfo.org > Arachis > hypogaea > genomes > Tifrunner.gnm2.J5K5), as described in previous studies [25]. The expression level of genes was presented as FPKM (fragments per kilobase per million reads) and calculated by RSEM (v1.3.1). Using the DESeq2 (v1.4.5), the differentially expressed genes (DEGs) were identified with a fold change ≥ 2 and a Q-value (adjusted *p*-value) ≤ 0.05 in different comparison groups, as in previous studies [24]. VENNY 2.1 (https://bioinfogp.cnb.csic.es/tools/venny/ (accessed on 10 May 2024)) was used to create the Venn diagrams, which exhibited the overlap of DEGs among different comparisons. To analyze the gene function, multiple databases, including NCBI non-redundant protein sequences (Nr), the Kyoto Encyclopedia of Genes and Genomes (KEGG) database, and Gene Ontology (GO), were used to annotate gene function by Phyper. Mfuzz (v2.6.0) (http://mfuzz.sysbiolab.eu (20 March 2024)) was used for K-means clustering analysis [26]. Bubble diagrams were prepared using TBtools-II (v2.031) software (https://github.com/CJ-Chen/TBtools/releases (accessed on 25 April 2024)) [27]. The size and color code of the bubbles represent normalized intensities of the FPKM values of the DEGs. The larger size of the bubble area, the higher the gene expression level, and the darker the color, the higher the gene expression level.

### 2.5. Validation of RNA-Seq Data Using qRT-PCR

The expressions of 17 randomly selected DEGs were chosen for validation the RNA-Seq results. The RNA samples used in RNA-Seq were also used for qRT-PCR, and reverse transcriptions were carried out using the PrimeScript II 1st Strand cDNA Synthesis Kit (TaKaRa, Dalian, China). The qRT-PCR reactions were carried out using the ABI7500 Real Time System (Applied Biosystems, Foster City, CA, USA) and the FastStart Universal SYBR Green Master (Roche, Indianapolis, IN, USA) according to the manufacturers’ protocol. Each PCR reaction was performed with a total volume of 20 μL containing 10 μL SYBR Green Master mix, 1 μL appropriately diluted cDNA, and 0.5 μM primers. The relative expressional levels of genes were calculated by the 2^−△△CT^ method according to previous studies [8]. The primers for each candidate gene were designed via Beacon Designer 8.0 software. The primers are listed in Supplementary Table S1.

### 2.6. Statistical Analysis

Statistical analysis was carried out via SigmaPlot Version 11.0, SPSS13.0, and Excel 2007 software.

## 3. Results

### 3.1. Mutant eg06g Acquisition and Phenotypic Analyses

A mutant library was constructed via ^60^Co γ-ray-radiating G06G, and an erect branching peanut mutant *eg06g* was identified. Compared with G06G, the average LBA of *eg06g* was 36.4° and significantly smaller than that of G06G (LBA 83.7°) (Figure 1A). 

### 3.2. Identification of Differentially Expressed Genes (DEGs) Between Upper Sides and the Lower Sides of Lateral Branch from eg06g and G06G 

To reveal the molecular mechanism underlying the formation of LBA in eg06g, RNA-seq was performed using the upper sides and the lower sides of the lateral branch from *eg06g* and G06G (Figure 1B), which were named EG06Gup, EG06GDown, G06Gup, and G06GDown, respectively. As shown in Table 1, each sample produced an average of about 22.10 M raw reads. After quality filtration, approximately 21.62 M clean reads remained per sample. Over 97.67% reads were mapped to the *A. hypogaea* L. reference genome, and over 81.38% reads were mapped to gene regions.

**Figure 2:**
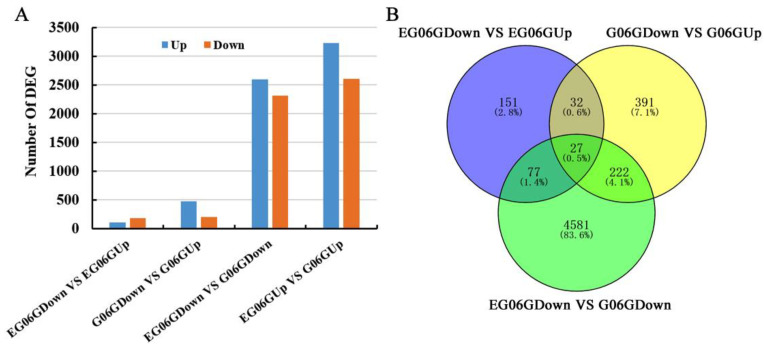
Statistical analysis of DEGs. (**A**) The numbers of DEGs in different comparison groups. (**B**) Venn diagrams presenting the overlap of DEGs among different comparisons.

In total, 58495 genes were identified (Table S2). According to the criteria of an absolute log2 fold change ≥ 1 and a *q*-value < 0.05, 108 upregulated DEGs and 179 downregulated DEGs were obtained between the lower sides and the upper sides of the lateral branch from *eg06g* (EG06GDown vs. EG06GUp). In G06G (G06GDown vs. G06GUp), 672 DEGs were identified, and 471 were upregulated and 201 were downregulated DEGs. There were 4908 DEGs and 5833 DEGs between the lower sides and the upper sides of the lateral branch of *eg06g* and G06G, respectively (Figure 2A and Table S3). The intersection of EG06GDown vs. EG06Gup with G06GDown vs. G06Gup represented the number of DEGs in *eg06g* and G06G between the lower sides and the upper sides of the lateral branch, which was 59, among which only 27 DEGs were shared in these lower sides of the branch between two materials (intersecting with EG06GDown vs. G06GDown) (Figure 2B). There were only 104 DEGs obtained from the intersection of EG06GDown vs. EG06GUp with EG06GDown vs. G06GDown (Figure 2B).

### 3.3. GO and KEGG Enrichment Analysis of DEGs

In order to gain insights into the function of DEGs, GO enrichment analysis was conducted. For the DEGs between the lower sides and the upper sides of the lateral branch from *eg06g*, GTP cyclohydrolase I activity, catalytic activity, oxidoreductase activity, and cellulose synthase activity were the top enriched GO terms of the molecular function, while the GO terms of biological process were mainly focused on the cellular polysaccharide metabolic process and the tetrahydrofolate biosynthetic process (Figure S1A and Table S4). For the DEGs from G06G, the most abundant terms of the molecular function were transcription cis-regulatory region binding, lipid binding, and oxidoreductase activity, while positive regulation of transcription by RNA polymerase II and positive regulation of biosynthetic process were the top terms in the biological process (Figure S1B and Table S4). In the DEGs of EG06GDown vs. G06GDown, oxidoreductase activity was the top term in the molecular function. In the biological process, DEGs were primarily distributed in terms of the carbohydrate metabolic process, cell wall organization or biogenesis, and the diterpenoid metabolic process (Figure S1C and Table S4). 

KEGG enrichment analysis showed that flavonoid biosynthesis, cutin, suberine and wax biosynthesis, and selenocompound metabolism were significantly enriched in the DEG of EG06GDown vs. EG06Gup (Figure S2A and Table S5). In G06G, the DEG between the upper sides and the lower sides of the lateral branch were mainly involved in glycerolipid metabolism, cutin, suberine and wax biosynthesis, other glycan degradation, and so on (Figure S2B and Table S5). The pathways related to flavonoid biosynthesis, MAPK signaling pathway, zeatin biosynthesis, and plant hormone signal transduction were enriched in the DEG between *eg06g* and G06G of the lower sides of the lateral branch (Figure S2C and Table S5), which indicates that plant hormones and signal transduction play major roles in the regulation of mutant phenotypes. 

### 3.4. K-Means Clustering of DEGs in Four Samples and Functional Enrichment Analysis

**Figure 3:**
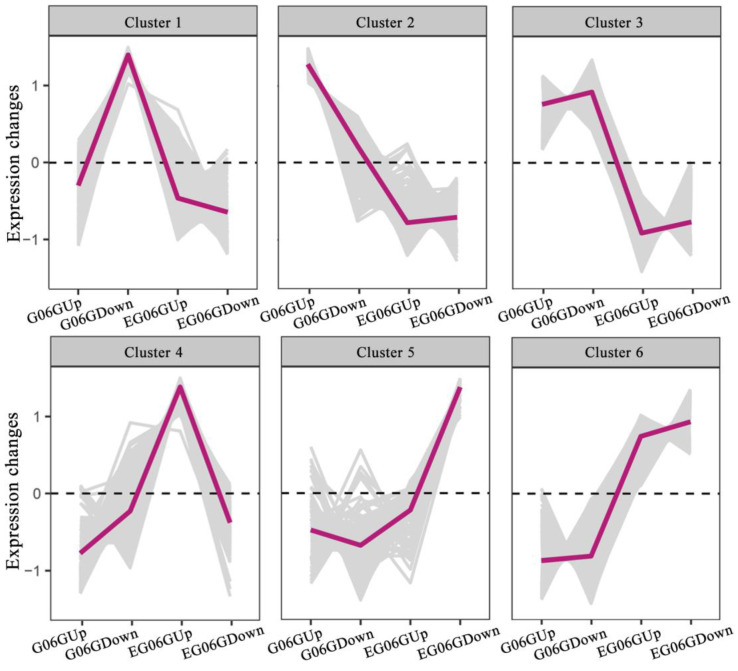
K-means clustering of gene expression profiles in the four samples (EG06Gup, EG06GDown, G06Gup, and G06GDown). The *x*-axis indicates the samples, and y-axis represents the relative expression level. Gray lines represent the relative expression levels in different samples, and the red line exhibits the average values of relative expression in each sub-cluster.

To investigate the regulatory processes that are critical for peanut branching habit, DEGs were clustered by their expression patterns across the four samples (EG06Gup, EG06GDown, G06Gup, and G06GDown). The results show that the majority of the genes could be grouped into six clusters (Figure 3 and Table S6).

DEGs in cluster 1 possessed a higher expression level in G06GDown, which is an integral component of the membrane and cellular macromolecule biosynthetic process and was enriched (Figure 3 and Table S6). Cluster 2 and Cluster 3 included 500 and 2386 DEGs with a higher expression level in G06G, respectively. These genes mainly participated in the metabolic process, solute carrier family, catalytic activity, sucrose synthase, cellulose synthase, and plant hormone signal transduction (Figure 3 and Table S6).

DEGs of sub-cluster 4, with a higher expression lever in EG06Gup, mainly enriched the carbohydrate metabolic process and plant hormone signal transduction (Figure 3 and Table S6). Cluster 5 contained 113 genes that were more abundant in EG06GDown and enriched in cellular component organization or biogenesis (Figure 3 and Table S6). DEGs in Cluster 6 had a higher expression level in *eg06g*, and these genes mainly represented biological functions related to cell wall modification, such as sucrose synthase, chitinase, xyloglucosyl transferase, pectinesterase, endoglucanase, cellulose synthase, xyloglucan O-acetyltransferase, and so on. The genes that participated in the plant hormone metabolic process and signal transduction were also enriched in this cluster (Figure 3 and Table S6). This result indicates that the enhanced cell wall metabolic process might contribute to the lateral branch transition from spreading growth to upright growth.

### 3.5. Starch and Sucrose Metabolism, Phytohormone Metabolism and Signaling Responsible for Phenotype of eg06g 

**Figure 4:**
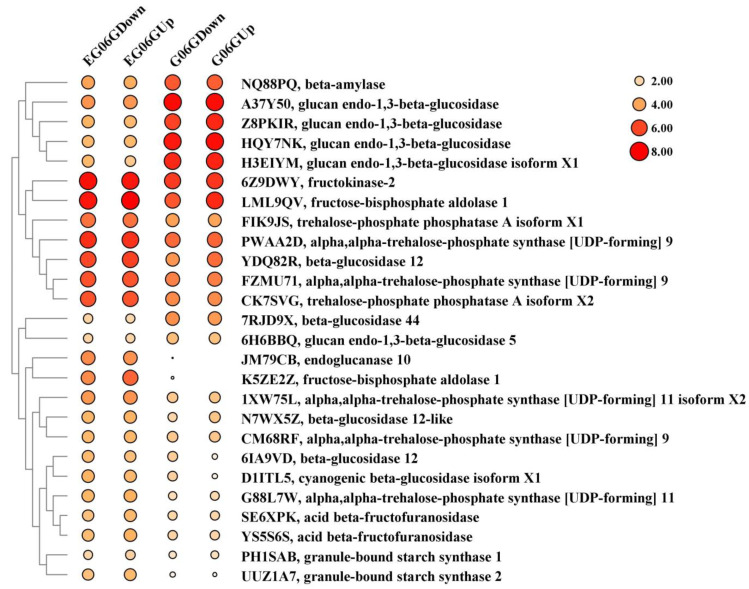
Bubble diagram of key DEGs related to starch and sucrose metabolism. The size and color code of the bubbles represent the normalized intensities of the FPKM (fragment per kilobase per million mapped) value of the DEGs in the four samples. The larger the bubble area, the higher the gene expression level, and the darker the color, the higher the gene expression level.

Starch-filled amyloplasts play a vital role in controlling shoot gravitropism and thus determining branch angle. Starch biosynthesis in amyloplasts influences the magnitude of gravitropic response and tiller angle [14]. A total of 26 DEGs encoding enzymes involved in starch and sucrose metabolism were identified between *eg06g* and G06G (Table S7 and Figure 4). The expression of two genes (PH1SAB and UUZ1A7) encoding granule-bound starch synthase, which is responsible for the synthesis of amylose, were upregulated in *eg06g*. β-Amylases (BAMs) catalyze the conversion of starch into maltose and play pivotal roles in controlling plant growth, development, and abiotic stress tolerance [28]. *BAM* (NQ88PQ) had a higher expression level in G06G compared with *eg06g* (Table S7 and Figure 4). In addition, two DEGs encoding fructose–bisphosphate aldolase, which is involved in the Calvin cycle, were dramatically upregulated in *eg06g*, especially at the lower sides and the upper sides of the lateral branch. These results indicate that the starch biosynthesis was enhanced in *eg06g*, which contributes to the sedimentation of amyloplasts and gravity perception at the base of the lateral branch.

**Figure 5:**
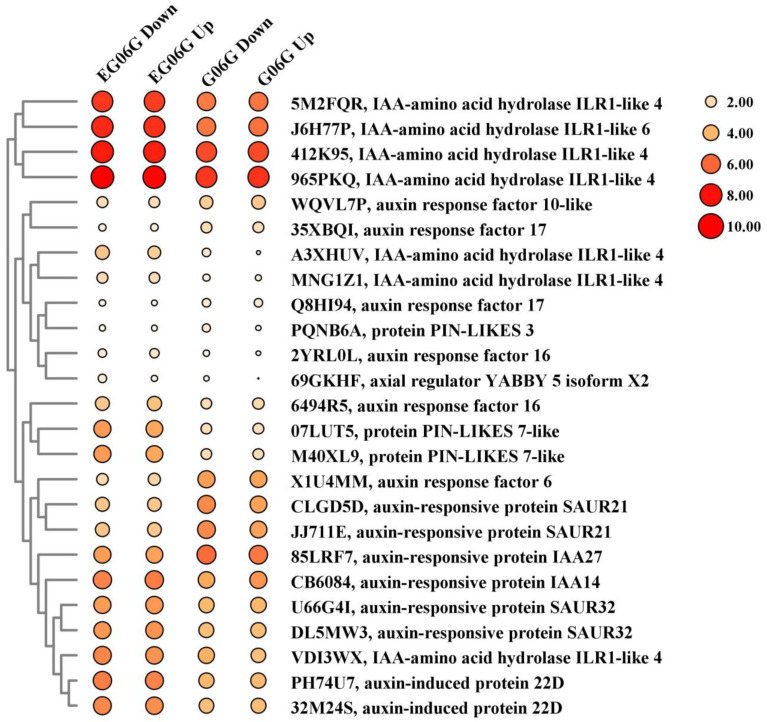
Bubble diagram of key DEGs related to auxin homeostasis and transport. The size and color code of the bubbles represent normalized intensities of the FPKM value of the DEGs in the four samples.

**Figure 6:**
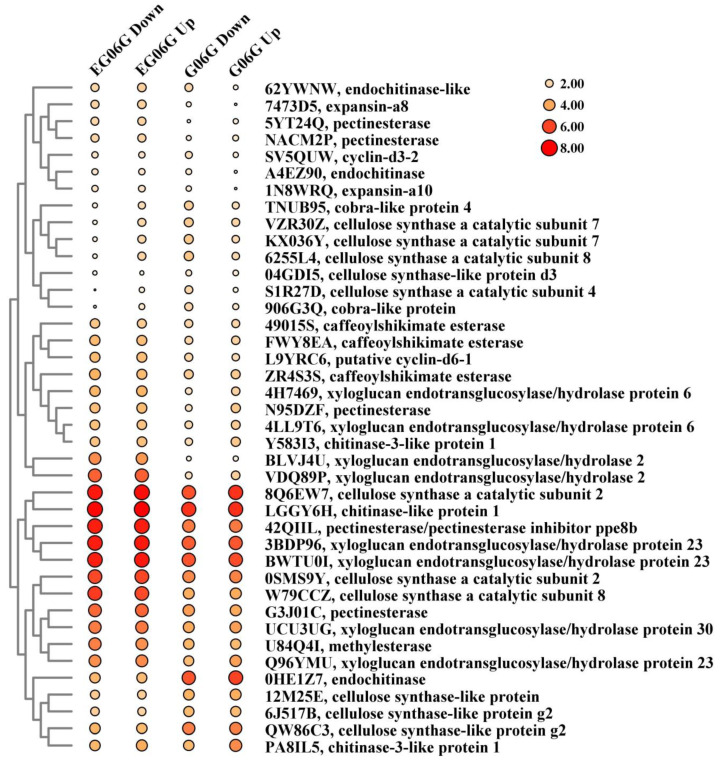
Bubble diagram of key DEGs involved in cell wall modification-related enzymes. The size and color code of the bubbles represent normalized intensities of the FPKM value of the DEGs in the four samples.

Seven genes encoding IAA-amino acid hydrolase ILR1-like protein, which catalyzes IAA-aa conjugates to free IAA and thereby regulate IAA levels, presented higher expression level in in *eg06g* (Table S7 and Figure 5). The auxin efflux carrier, PIN proteins, are mainly responsible for mediating auxin gradient, which was critical for the tiller/branch angle. Two genes (M40XL9, 07LUT5) encoding PIN-LIKES proteins were upregulated in *eg06g* (Table S7 and Figure 5). The early auxin-induced genes, two auxin response factors (6494R5, 2YRL0L), one auxin-responsive IAA14 protein (CB6084), and two auxin-responsive SAUR32 proteins (U66G4I, DL5MW3) had higher expression levels in *eg06g* compared with those in G06G. In addition, one auxin-responsive IAA27 protein (85LRF7) and two auxin-responsive SAUR21 proteins (CLGD5D, JJ711E) showed higher expression levels in G06G than those in *eg06g* (Table S7 and Figure 5). Auxin promotes cell division, growth (expansion, elongation), and differentiation via acidification of the cell wall, which activates cell wall modification-related enzymes [29,30]. In our results, cell wall-related genes, such as caffeoylshikimate esterase, cellulose synthase, pectinesterase, xyloglucan endotransglucosylase/hydrolase, and expansin, were significantly upregulated in *eg06g* (Table S7 and Figure 6). 

Cytokinin hydroxylase and cytokinin dehydrogenase take part in cytokinin biosynthesis and degradation, respectively. Zeatin O-glucosyltransferase is responsible for control of cytokinin glucosylation levels and balancing cytokinin homeostasis. In *eg06g*, two genes (24HCRC, 84B366) encoding cytokinin hydroxylase and two genes (BDKN1M, P44SU2) encoding zeatin O-glucosyltransferase were significantly upregulated compared with those in G06G. In contrast, two genes encoding cytokinin dehydrogenase (E69B57, 22S773) exhibited lower expression levels in *eg06g* (Table S7 and Figure S3). GA biosynthesis and deactivation is tightly controlled by gibberellin 20 oxidase and Gibberellin 2-β-dioxygenase, respectively [31]. Genes encoding gibberellin 20 oxidase were identified. Among them, the expression of three genes (2861FR, F7AUT2, 4TCZ1X) were upregulated in G06Gup and G06GDown (Figure S4). Inversely, the expression of five gibberellin 2-β-dioxygenase genes were downregulated in G06Gup and G06GDown (Table S7 and Figure S4). 

### 3.6. Validation of DEGs Using qRT-PCR

**Figure 7:**
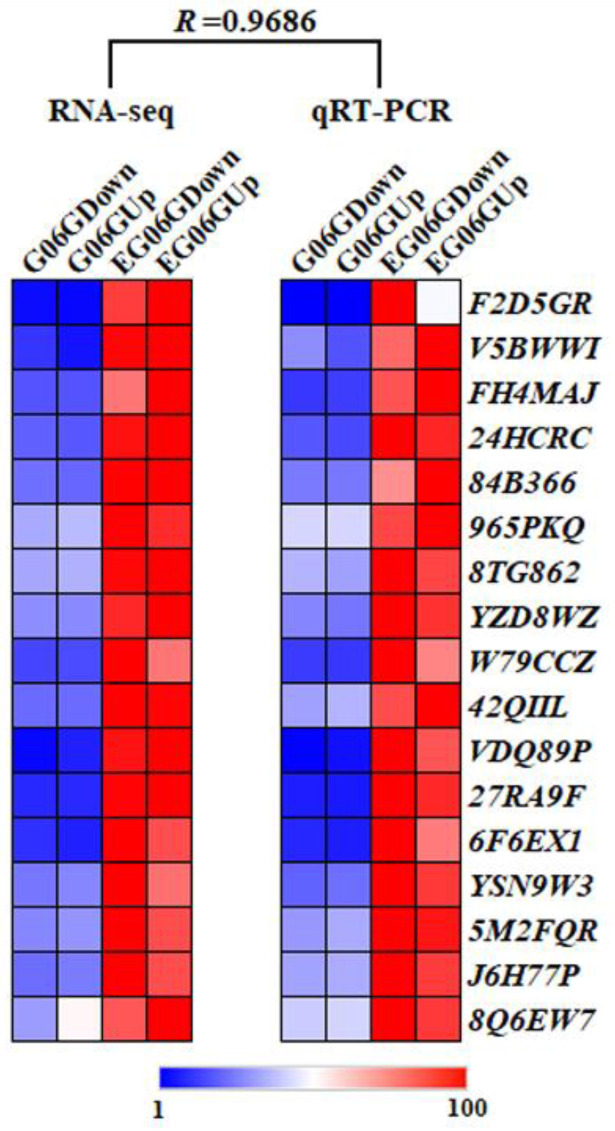
qRT-PCR verification of selected DEGs in *eg06g* and G06G. R represents the correlation coefficient between RNA-seq data and qRT-PCR.

To validate the RNA-seq data, 17 DEGs related to phytohormone metabolism, signal transduction, and cell wall modifications were randomly selected, and qRT-PCR was conducted (Figure 7). The results exhibit similar expression patterns, and the correlation coefficient was 0.9686 between qRT-PCR with RNA-seq (Figure 7), which confirms the creditability and repeatability of the data.

## 4. Discussion

The molecular mechanism of the tiller angle and the branch angle has been intensively researched in rice and *Arabidopsis*. However, the regulation basis of the peanut branching habit is just beginning. In our research, an erect branching peanut mutant, *eg06g*, was identified from the mutant library of the ^60^Co γ-ray-radiating spreading-type peanut cultivar G06G. The average LBA of *eg06g* was only 36.4°, which was significantly smaller than that of G06G (LBA 83.7°) (Figure 1A). This provides valuable materials for studying the molecular basis underlying peanut growth habit. 

Auxin homeostasis, transport, and gradients play major roles in regulating organ formation, growth, shoot gravitropism, and, ultimately, plant shape [14,32]. In our result, the expression of auxin transport-related genes, such as *PIN-LIKES*, was found to be increased in *eg06g* (Table S7 and Figure 5). In *Arabidopsis*, five PINs mediate directional auxin flow and maintain auxin gradient distribution [33]. Functional analyses demonstrated that OsPIN1a and OsPIN1b redundantly controlled root gravitropism and the tiller angle. The *pin1a pin1b* double mutant displayed an increased tiller number and a larger tiller angle and panicle branch angle [34]. In switchgrass, PvPIN1 participated in regulating the auxin-dependent tiller angle and tiller number [35]. PLANT ARCHITECTURE AND YIELD 1 (PAY1) improved plant architecture through affecting basipetal IAA transport and altering endogenous IAA distribution [36]. Tiller Angle Control 4 (TAC4) regulated rice shoot gravitropism and the tiller angle via affecting the auxin content and distribution. In the coleoptiles of the *tac4* mutant, the expression of IAA synthetic genes such as *YUC5*-*7* and *TAR2* was attenuated [37]. Seven genes related to IAA metabolism were also upregulated in *eg06g* (Table S7 and Figure 5). Correspondingly, auxin-induced genes, such as auxin response factors *IAA14* and *SAUR32*, were upregulated in *eg06g*. The upregulated auxin transport- and metabolism-related genes might have contributed to altering the auxin polar auxin transport (PAT) and gradient distribution in *eg06g*, which resulted in the erect branching phenotype. 

Auxin-mediated cell expansion has been identified to play a crucial role in diverse aspects of plant growth and development, such as tropic bending, organ growth, and shoot elongation in response to light and temperature cues [38]. High auxin concentrations upregulated *SAUR* genes that bind and inactivate PP2C.D phosphatase activity, which activates plasma membrane (PM) H^+^-ATPases and results in apoplastic acidification and PM hyperpolarization [39]. Apoplast acidification activates cell wall modification-related enzymes, including expansins, pectin methylesterases (PME), cellulases, and xyloglucan endotransglycosylase/hydrolases (XTHs) [29,30,38]. Auxin-induced organ formation requires a decrease in cell wall rigidity via controlling PME activity [40]. The enzyme activities of XTHs in growing tissue are sensitive to apoplastic pH. Auxin decreased the pH value by about 1 unit in several plant tissues, which resulted in upregulated XTH activity. The XTH was responsible for cutting xyloglucan (XyG) backbones and providing short XyG fragments, which resulted in the loosening of the wall and impetus of the wall rearrangement for cell elongation [30]. In *eg06g*, the upregulated *SAUR32* genes might have been responsible for apoplast acidification, and then triggered cell wall relaxation and cell elongation. Correspondingly, the expression of cell wall modification-related genes, such as Caffeoylshikimate esterase, cellulose synthase, pectinesterase, xyloglucan endotransglucosylase/hydrolase, and expansin, were upregulated in *eg06g* (Table S7 and Figure 6). The elongation of the lateral branch in *eg06g*, especially at the lower sides, was conductive to the transition from spreading growth to upright growth.

Cytokinins are included in a variety of plant growth processes—for example, cell division, photosynthesis, chloroplast differentiation, regulation of leaf senescence, and maintenance of meristem function, especially in shoots and roots [40]. A higher concentration ratio of cytokinin to auxin facilitates shoot development, and a lower ratio of cytokinin to auxin promotes root development [41]. Cytokinins promote the accumulation of PIN3, PIN4, and PIN7 on the PM and alter auxin transport, which contributes to increased shoot branching [22]. The degradation of cytokinins could be triggered by cellulose biosynthesis inhibition and thus inhibit cellular proliferation in the root tissues [42]. During gravitropic responses, GA showed asymmetric action. Asymmetric auxin distribution, which is instructive for gravity-induced differential growth, is consolidated by the asymmetric action of GA via stabilizing the PIN-dependent auxin stream [21]. In our results, the DEGs coding cytokinin and GA biosynthetic and metabolic enzymes were also identified between *eg06g* and G06G (Table S7, Figures S3 and S4). These results demonstrate that auxin, cytokinin, and GA work synergistically to finely regulate the formation of peanut lateral branch angles.

## 5. Conclusions

In this study, an erect branching peanut mutant, *eg06g*, was obtained via ^60^Co γ-ray-radiating treatment of G06G. Comparative transcriptomes between the upper sides and the lower sides of the lateral branch from *eg06g* and G06G were performed. Genes related to starch biosynthesis were enhanced in *eg06g*, which might have improved amyloplast sedimentation. Auxin homeostasis- and transport-related genes were found to be upregulated in *eg06g*, and correspondingly, the expression of auxin-responsive protein also increased. Auxin gradient distribution mediates cell expansion via apoplast acidification to activate cell wall modification-related enzymes, and then triggers cell wall relaxation and cell elongation. Ultimately, the cell elongation of lateral branch was conductive to the transition from spreading growth to upright growth. In addition, cytokinin and GA also took part in controlling this process, and together with auxin, finely regulated the formation of peanut lateral branch angles. Our research presents a critical landscape for further deciphering the molecular mechanism of peanut LBA formation and provides new germplasm resources for breeding peanut cultivars with ideotypes.

## Figures and Tables

**Table 1:** Summary of the total read numbers obtained from each sample of *eg06g* and G06G.

Sample	Total Raw Reads (M)	Total Clean Reads (M)	Total Clean Bases (Gb)	Clean Reads Q20 (%)	Clean Reads Q30 (%)	Clean Read Ratio (%)	Total Genome Mapping (%)	Uniquely Genome Mapping (%)	Total Gene Mapping (%)	Uniquely Gene Mapping (%)
EG06GDownA	21.59	21.35	1.07	97.88	91.43	98.88	98.26	44.18	82.31	11.75
EG06GDownB	21.59	21.07	1.05	97.7	91.18	97.6	97.33	43.86	81.54	11.76
EG06GDownC	21.59	21.12	1.06	97.88	91.6	97.83	98.04	44.04	82.23	11.73
EG06GUpA	21.59	21.33	1.07	97.89	91.5	98.79	98.17	44.22	82.06	11.78
EG06GUpB	23.75	23.12	1.16	97.73	91.13	97.36	97.87	44.18	81.68	11.83
EG06GUpC	21.59	21.09	1.05	97.91	91.69	97.69	98.03	44.2	81.96	11.81
G06GDownA	21.59	21.26	1.06	97.92	91.69	98.47	97.51	44.24	80.71	11.64
G06GDownB	23.75	23.12	1.16	98.07	92.31	97.35	97.59	44.22	80.95	11.61
G06GDownC	23.75	22.98	1.15	97.96	91.94	96.75	97.6	44.23	80.96	11.63
G06GUpA	21.59	21.23	1.06	98.04	92.09	98.33	97.32	44.02	80.82	11.63
G06GUpB	21.59	21.06	1.05	97.92	91.76	97.57	97.16	44	80.59	11.63
G06GUpC	21.19	20.75	1.04	97.87	91.52	97.94	97.13	43.94	80.73	11.62
Average value	22.10	21.62	1.08	97.90	91.65	97.88	97.67	44.11	81.38	11.70

## Data Availability

The original contributions presented in the study are included in the article/Supplementary Materials, further inquiries can be directed to the corresponding authors.

## Supplementary Materials

The following supporting information can be downloaded at: https://www.mdpi.com/article/10.3390/genes15101348/s1, Table S1. Oligonucleotide primers used in qRT-PCR. Table S2. Expression profiles and gene annotations of DEGs in RNA-seq. Table S3. List of DEGs in each comparison group. Table S4. GO enrichment analysis of DEGs in each comparison group. Table S5. KEGG enrichment analysis of DEGs in each comparison group. Table S6. List of DEGs in each sub-cluster of K-means. Table S7. Key genes responsible for phenotype of *eg06g*. Figure S1. Bubble diagram of top 20 enriched GO terms of DEGs in each comparison group. Figure S2. Bubble diagram of top 20 enriched KEGG pathways of DEGs in each comparison group. Figure S3. Bubble diagram of key DEGs related to cytokinin biosynthetic and metabolic enzymes. The size and color code of the bubbles represent normalized intensities of the FPKM value of the DEGs in the four samples. Figure S4. Bubble diagram of key DEGs involved in GA biosynthetic and metabolic enzymes. The size and color code of the bubbles represent normalized intensities of the FPKM value of the DEGs in the four samples.
