# Supplementary material for: Transcriptome Analysis Deciphers the Underlying Molecular Mechanism of Peanut Lateral Branch Angle Formation Using Erect Branching Mutant
Source: Genes (Basel). 2024 Oct 21;15(10):1348. doi: 10.3390/genes15101348 (PMC11507551; doi:10.3390/genes15101348)
Supplement: Supplementary file 1 [file genes-15-01348-s001.zip › Table S1.pdf]

Table S1 List of the sequences of the primers used in qRT-PCR.

Gene ID	Protein	Primer	Sequence (5'-3')	Annealing temperature	Amplicon size
F2D5GR	ethylene-A1esponsive transcription factor ERF113	F2D5GR-S1	CATCCTCTGCTTCTATTC	55℃	132bp
		F2D5GR-A1	TCTCCTCATCATTCTTCT		
V5BWWI	gibberellin 2-beta-dioxygenase 1	V5BWWI-S1	TCTCTTCTTCTTCTTCTTCTTCTT	55℃	101bp
		V5BWWI-A1	GGCTGCTCTGTTATTGCTA		
FH4MAJ	gibberellin 2-beta-dioxygenase 2	FH4MAJ-S1	TCCACCAACAACAAGATT	55℃	143bp
		FH4MAJ-A1	GCAAGACTATGAACAATGAG		
24HCRC	cytokinin hydroxylase	24HCRC-S1	TTGGTAGGAACTTGGCATT	55℃	100bp
		24HCRC-A1	GTTGGTGAGTGACGGTAA		
84B366	cytokinin hydroxylase-like	84B366-S1	ATCTTCCTCATCATCATC	55℃	151bp
		84B366-A1	TATTCTCTTCAACAACTCT		
965PKQ	IAA-amino acid hydrolase ILR1-like 4 isoform X1	965PKQ-S1	ATAGTATTAGCACCTACC	55℃	149bp
		965PKQ-A1	TATGATGAACAAGTTGAC		
8TG862	sugar carrier protein C	8TG862-S1	CTTCATCTACTTCTTCCT	55℃	112bp
		8TG862-A1	GCCATAATCATCATTCTC		
YZD8WZ	sugar carrier protein C	YZD8WZ-S1	CCATCTTCATCTACTTCT	55℃	137bp
		YZD8WZ-A1	CTTATTCAACTCAACTTCA		
W79CCZ	cellulose synthase A catalytic subunit 8	W79CCZ-S1	ACCAAGACCATAACCAAGAA	55℃	98bp
		W79CCZ-A1	TCCATTAGCATCAACTCCAA		
42QIIL	pectinesterase/pectinesterase inhibitor PPE8B	42QIIL-S1	AACAGAGCCACTAATAATAATAC	55℃	121bp
		42QIIL-A1	GAGAAGCATCAACAAGAAG		
VDQ89P	xyloglucan endotransglucosylase/hydrolase 2 isoform X1	VDQ89P-S1	AACAACAATGACAACACTA	55℃	141bp
		VDQ89P-A1	ATCTTAGCACGGTTATCA		
27RA9F	serine/threonine-protein kinase CST	27RA9F-S1	TCAAGAGTGGAAGTCAGA	55℃	102bp
		27RA9F-A1	ACAAGGAGAAGTTCATCAG		
6F6EX1	serine/threonine-protein phosphatase 7 long form homolog	6F6EX1-S1	TACCTCATTACGACCTTA	55℃	117bp
		6F6EX1-A1	TATCATCCTCATTGTTACC		

YSN9W3	ethylene-A1esponsive transcription factor RAP2-7 isoform X1	YSN9W3-S1	CATTGGTCGCATAATAGCA	55℃	102bp
		YSN9W3-A1	AAGAACAGACGCATCAGA		
5M2FQR	IAA-amino acid hydrolase ILR1-like 4	5M2FQR-S1	TCATCATCATCATCATCACT	55℃	86bp
		5M2FQR-A1	CTCCTTATTCTCTTCATCCA		
J6H77P	IAA-amino acid hydrolase ILR1-like 6 isoform X1	J6H77P-S1	ATCATCATCATCATCATCATCAC	55℃	89bp
		J6H77P-A1	TCCTTATTCTCTTCATCCACTT		
8Q6EW7	cellulose synthase A catalytic subunit 2	8Q6EW7-S1	AGAGGATGGTGTTGATGA	55℃	95bp
		8Q6EW7-A1	TAAGGCGAGAAGATAGCA		
AhActin		AhActin-S1	GTCATCGTCATCCTCTTCTC	55℃	
		AhActin-A1	CATTCCTGTTCCATTGTCAC		
