# Supplementary figures and images for: Transcriptome Analysis Deciphers the Underlying Molecular Mechanism of Peanut Lateral Branch Angle Formation Using Erect Branching Mutant
Source: Genes (Basel). 2024 Oct 21;15(10):1348. doi: 10.3390/genes15101348 (PMC11507551; doi:10.3390/genes15101348)

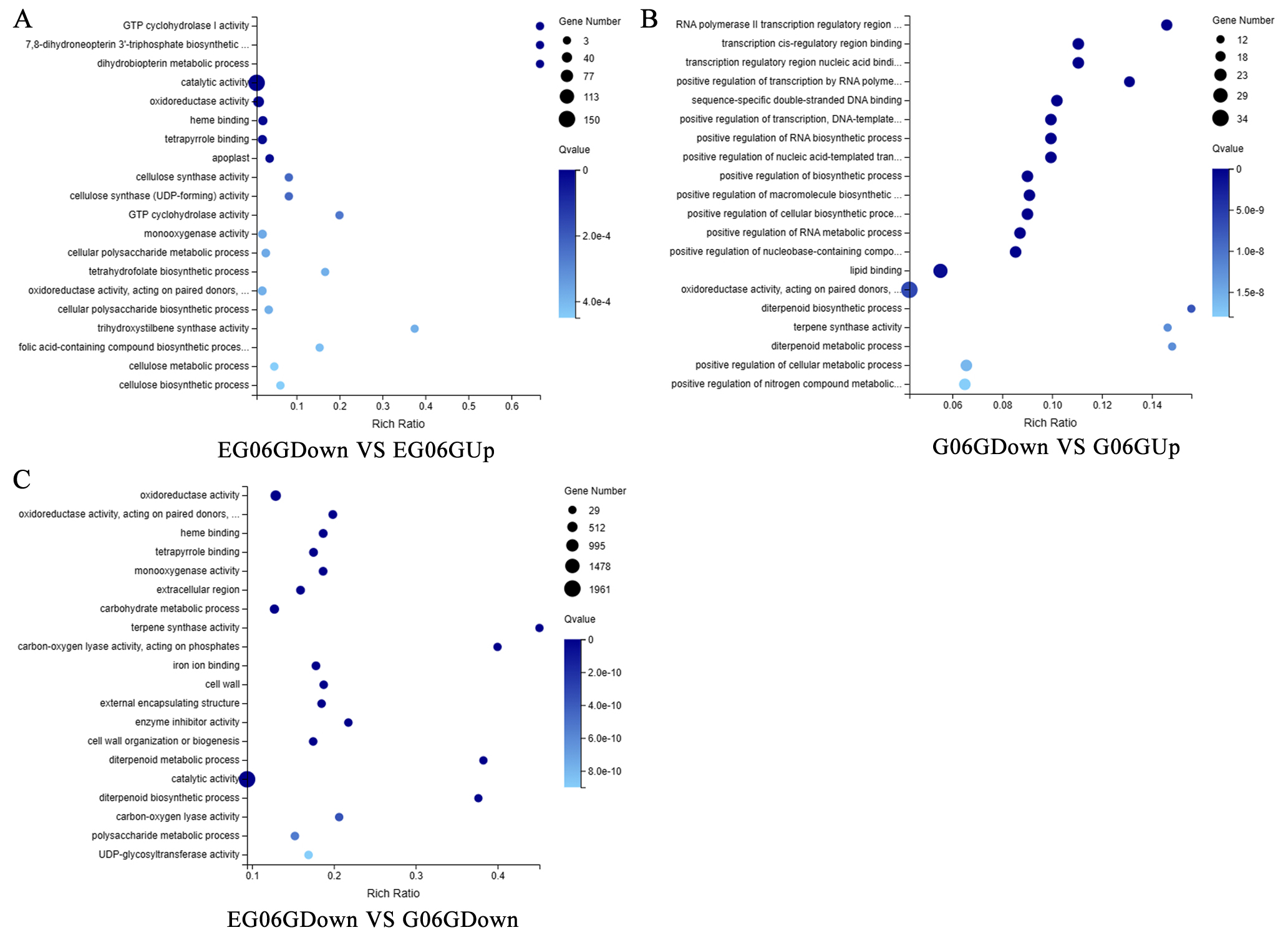

Supplement: Supplementary file 1 [file genes-15-01348-s001.zip › Figure S1.jpg]

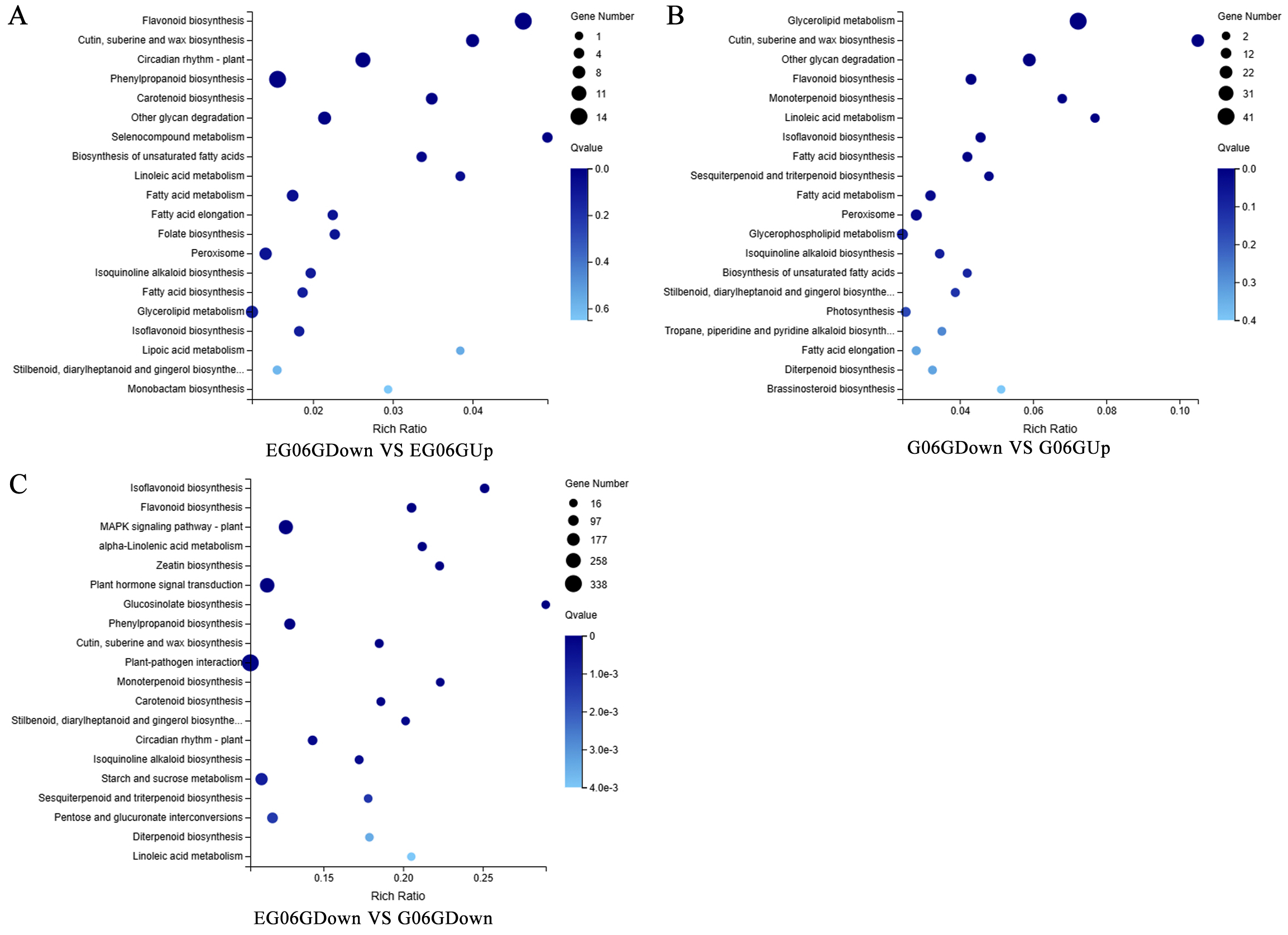

Supplement: Supplementary file 1 [file genes-15-01348-s001.zip › Figure S2.jpg]

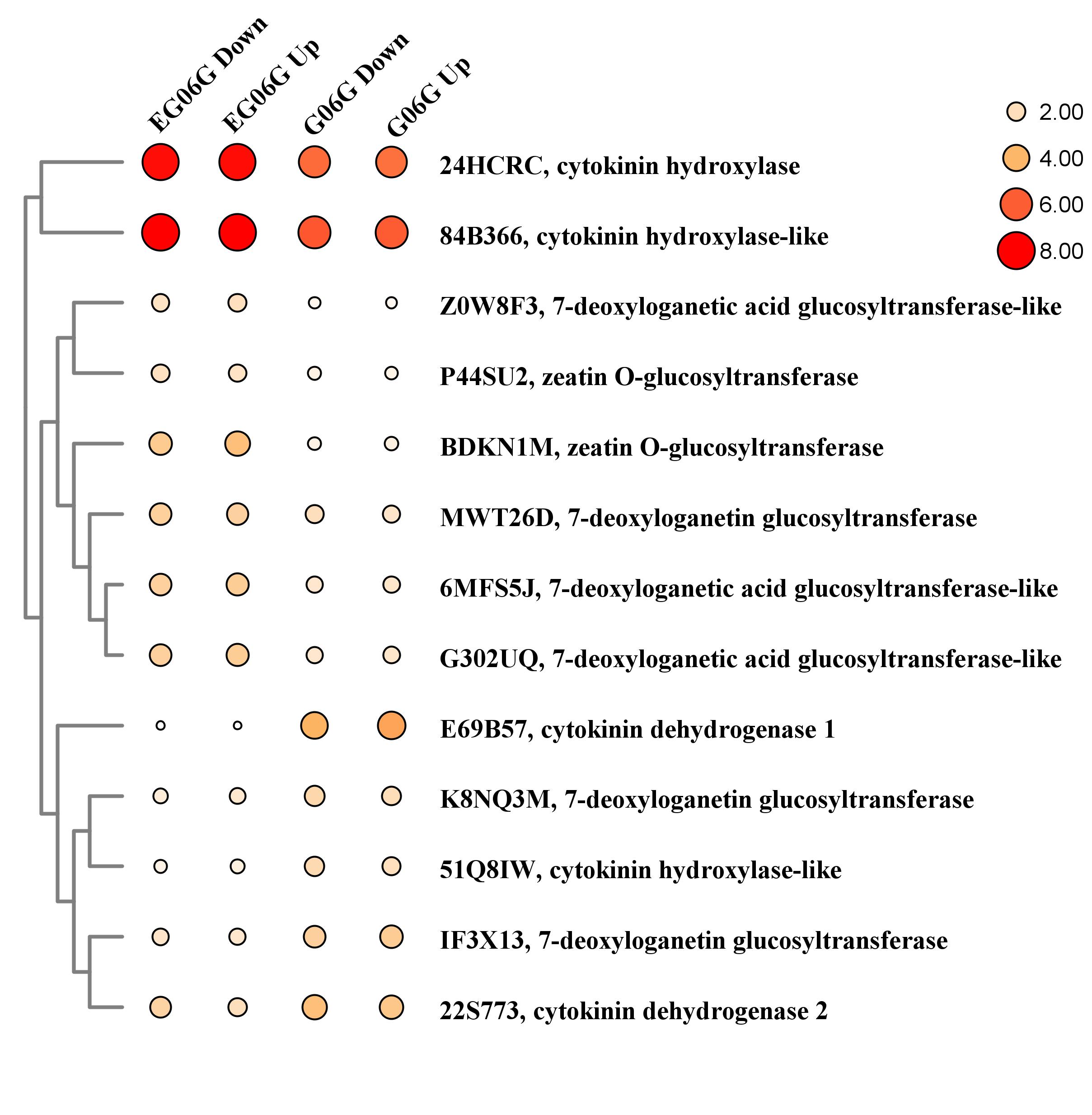

Supplement: Supplementary file 1 [file genes-15-01348-s001.zip › Figure S3.jpg]

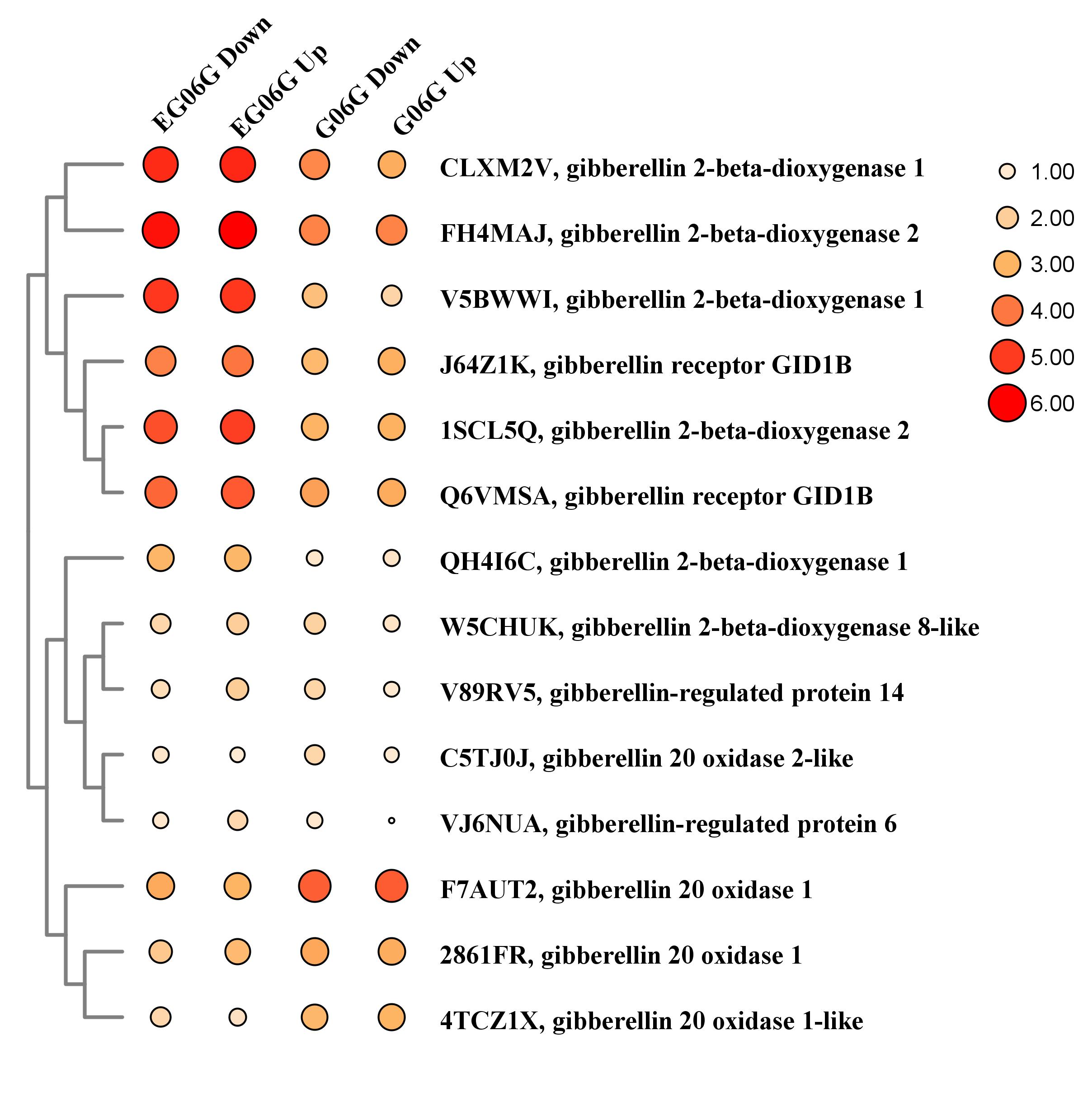

Supplement: Supplementary file 1 [file genes-15-01348-s001.zip › Figure S4.jpg]
